# A Sieve-Raft Hypothesis for the regulation of endothelial fenestrations

**DOI:** 10.5936/csbj.201308003

**Published:** 2013-08-24

**Authors:** Victoria C. Cogger, Ute Roessner, Alessandra Warren, Robin Fraser, David G. Le Couteur

**Affiliations:** a Centre for Education and Research on Ageing and ANZAC Research Institute, Concord Hospital and University of Sydney, Sydney NSW, Australia; b Charles Perkins Centre, University of Sydney NSW Australia; c Metabolomics Australia and Australian Centre for Plant Functional Genomics, The University of Melbourne, 3010 Victoria, Australia; d Christchurch School of Medicine, University of Otago, Christchurch NZ

Fenestration morphology is a remarkable example of the synergy between structure and function. Through a better understanding of fenestration structure our understanding of its function will be enhanced.

Fenestrations are transcellular pores that act as fundamental biological ultra-filters allowing diffusive and convective passage of substrates across cells without relying on endocytosis or other receptor-mediated mechanisms. They facilitate passive transfer of substances such as lipoproteins [1], parasites [2], pharmacological agents [3] and gene transfer vectors [4]. Fenestrated cells are highly conserved in evolution and have been documented in all species from fish to humans [5–9] and even in the phloem vascular system of higher plants [10]. In animals they are found in several cell types including liver sinusoidal endothelial cells (LSECs) [11] (Figure 1), glomerular endothelial cells [5], endothelial cells of the area postrema [12] and the posterior pituitary [13] of the brain, as well as numerous cancers [14]. All of these tissues require unimpeded transfer of substances between blood and surrounding cells. Fenestrations are essential for human health and loss of fenestrations in LSECs results in impaired lipid, drug and insulin transfer [15–17] and regeneration [18]. However, despite their ubiquity and biological importance, we are only beginning to understand the molecular and cellular pathways, and the spatial and temporal sequence of events involved in fenestration formation. Here, we propose a novel sieve-raft hypothesis [19] as a key mechanism regulating fenestrations in the LSEC.

**Figure 1:**
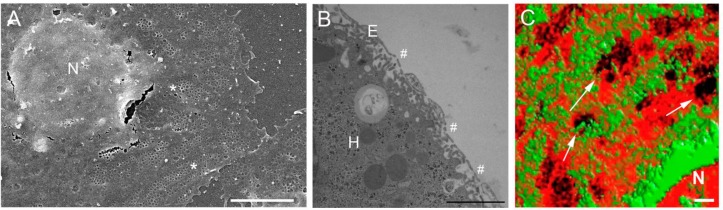
Microscopy of LSEC fenestrations and the LSEC membrane. Figure 1A is a scanning electron micrograph of an isolated LSEC in culture. The micrograph clearly displays fenestrations, examples are denoted by an asterix (*), arranged in groups (sieve plates) or individually. The fenestrations are located in the thin cytoplasmic extensions of the cell, distal to the nucleus (N) Scale bar = 5 µm. Figure 1B is a transmission electron micrograph of perfusion fixed liver, the unique architecture of the sinusoid can be seen. The very thin endothelium (E) is perforated with fenestrations (#), allowing passage of substrates into the hepatocytes (H) for metabolism, storage and detoxification Scale bar = 2 µm. Figure 1C is a micrograph prepared by 3D structured illumination microscopy. The LSECs have been stained with Bodipy FL C5 ganglioside GM1, a marker for rafts (green) and Cell-Mask Orange, a cell membrane marker (orange). There is an inverse distribution between liver sieve plates and membrane rafts. Some sieve plates are identified by an arrow (→) and fenestrations can be resolved within the sieve plates. Scale bar = 1 µm.

## Liver Sinusoidal Endothelial Cells (LSECs)

LSECs line the liver sinusoids which form the reticulated network of blood vessels of the highly vascular liver. The fractal dimension of the sinusoidal vessels (a measure of complexity) exceeds two indicating the space-filling characteristic of the sinusoids [20]. This degree of vascularity facilitates the exchange of substrates between blood and the liver and provides an extensive endothelial surface area for interactions with circulating immune cells and various colloid and soluble macromolecular waste products. The morphology of LSECs further facilitates cellular interactions and transfer of material from the blood through the presence of fenestrations which are between 50 and 200 nm in diameter and too small to be observed with conventional light microscopy. They are mostly found in attenuated areas of the cell cytoplasm, typically less than 100 nm in thickness. Fenestrations are bound by the plasma membrane and are discrete regions of fusion of the apical and basolateral membranes of the cell. They are complete gaps in the endothelial lining, lacking either a diaphragm or underlying basal lamina. In the LSEC, fenestrations are either scattered individually across the endothelial surface or are arranged in groups of between 10 and 100 fenestrations, termed ‘liver sieve plates’, reflecting their role as a filter or sieve [21] (Figure 1A). There are approximately 3-20 fenestrations per µm^2^ of endothelial surface and between 2-20% of the surface of the LSEC are covered by fenestrations [6, 22–27]. Between 60-75% of fenestrations are found within sieve plates in rats [22]. Sieve plates are particularly apparent in healthy young liver endothelial cells and are decreased with actin disruptors such as cytochalasin B [13]. In isolated LSECs, there are usually tens of sieve plates present in the cytoplasmic extension of a single cell, representing many hundreds or even thousands of fenestrations per cell [28, 29]. Fenestrations have been detected using a variety of methodologies (transmission electron microscopy, scanning electron microscopy, electron tomography, freeze fracture microscopy, cryo-electron microscopy, atomic force microscopy, and structured illumination microscopy [23, 24, 30–32]) (Figure 1). Even so, the exact size and morphology of fenestrations are difficult to measure [30].

## Biological function of fenestrations

The fenestrated LSEC acts as a filter and hence was termed ‘the liver sieve’ [33–35]. In the liver fenestrations permit the passage of a wide range of substrates (plasma and substrates within plasma, plasma proteins including albumin, smaller lipoproteins, colloidal particles and polystyrene microspheres) into the underlying space of Disse although the proportion of each substrate that enters the space of Disse via fenestrations remains unknown [36]. The diaphragmed fenestrations of the kidney facilitate the movement of water and other dissolved substances movement from the blood into the Bowmans capsule to produce urine [37].

Both diameter and frequency of fenestrations determines diffusive and convective transfer across the LSEC [38]. It is possible to quantify the effects of changes in the liver endothelium and fenestrations on the transfer of substrates such as lipoproteins by application of the engineering principles related to membrane filtration, specifically ultrafiltration. The reduction in the diameter of fenestrations will also influence the size of particles that are able to transfer across the endothelium. A summary of the physiological roles the fenestrations of the LSEC are listed in Table 1.

**Table 1:** A summary of the physiological roles of Fenestrations in LSECs.

Physiological roles of fenestrations in LSECs	
	**References**
The transfer of lipoproteins, particularly chylomicron remnants	[1, 40–42]
Transfer of soluble and protein bound substrates such as paracetamol, diazepam etc	[3, 43, 44]
Trans-endothelial hepatocyte-lymphocyte interactions (TEHLI) and inflammatory cell transfer	[45–47]
Vascular resistance	[48, 49]
Transfer of pathogens (malaria, hepatitis, gene therapy)	[2, 4, 50, 51]
Formation of lymph	

There are numerous reports of diseases and pathological processes that influence fenestrations, including: liver disease [42, 48, 49], liver toxins [50–53], [54, 55], systemic disease [56, 57], and other liver processes such as aging [36, 58–60], These changes have not usually been diagnostic [61, 62] but the overall trends are that: (1) acute toxic injury and acute medical conditions are associated with loss of endothelial integrity characterized by gap formation and (2) sub-acute and chronic conditions have been associated with defenestration and reduced porosity.

Age-related pseudocapillarization is now well documented [58, 63, 64]. This loss of endothelial fenestrations, endothelial thickening and increased deposition of extracellular matrix with age has been shown to impact upon liver function, in particular leading to a reduction in the transfer of lipids and pharmaceutical agents [1, 3, 65]. Through this mechanism age-related pseudocapillarization is thought to contribute to the development of age-related diseases.

## Fenestration regulation and dynamics

Fenestrations are dynamic structures that change in frequency and diameter in response to numerous stimuli in vitro. In vivo it is likely that fenestrations open and close in response to various stimuli such as inflammation, dietary fat load and/or circulating vasoactive cytokines and hormones (Table 2) [66, 67]. Local paracrine and autocrine factors presumably establish and maintain porosity at a level required for health. There are several issues that confound the interpretation of studies of regulatory factors. LSECs isolated from rat livers have been the major model for studying fenestration biology. This is dependent on the methodology with some methods failing to generate well fenestrated cells [29, 68, 69]. Isolated LSECs are only viable for 1-2 days and there is a dramatic change in fenestrations during this period [68, 70–73]. Maintenance of fenestrations in isolated LSECs requires VEGF [28, 68, 70] and extracellular matrix derived from the liver [73, 74]. It is likely that fenestrations are regulated in vivo by a variety of paracrine and circulating factors as well as the extracellular matrix and of course these are absent in isolated cell studies [69, 70].

**Table 2:** Fenestration active agents. Fenestration number and size can be modulated by numerous substances in vivo and in vitro. A number of these substances are listed below.

Treatment	Diameter of fenestrations	Number of fenestrations per cell
**Actin disruptors**		
Cytochalasin B	↑↓	↑
Dihydrohalichondramide	↓	↑
Latrunculin A	↓	↑
Misakinolide	↓	↑
Swinholide A	↓	↑
**Other**		
Acetylcholine	↑	?
Adrenaline	↓	?
Bethanechol	↑	?
Calmodulin agonist W-7	↑	?
Carbon tetrachloride	↑	↓
Cocaine and ethanol	?	↓
Collagen IV	n.c.	↑
Diethyl nitrosamine	?	↓
Dimethyl nitrosamine	n.c.	↓
DOI (2,5-dimethoxy-4-iodoamphetamine)	↑	↑/n.c.
Endothelin 1	↓	↓
ET_A_-R antagonist (BQ123)	↑	?
Ethanol acute dose	↑	↓
Ethanol chronic dose	↑↓	↓
Fatty liver	?	↓
Hypoxia	↑	?
Hepatectomy	↑	↓
Hepatitis C	↓	↓
Ionophore A23187	↓	?
Irradiation	↑	?
Isoproterenol	↑	?
Jasplakinolide	↓	↑
Laminin	n.c.	↓
Neuropeptide Y	↓	?
Noradrenaline	↓	?
Nicotine	↓	?
Pantethine	↑	↑
Phalloidin	↑	?
Phorbol myristate acetate	n.c.	↑↓
Pressure	↑	?
Prostaglandin E_1_	↑	?
Serotonin	↓	?
Temperature 4 °C	?	↓
Thioacetamide	↓	↓
Tumor cells	↓	↓
TNF-α	?	↓
Vasoactive intestinal peptide	↑	?
Vascular Endothelial Growth Factor	↑	↑

Legend:↑ = increase, ↓ = decrease, ↑↓ = conflicting reports, n.c. = no change,? = unknown

## Conventional wisdom on fenestrations

It is widely accepted that the actin cytoskeleton has a role in maintaining fenestrations. Sieve plates are supported by the actin cytoskeleton with structures such as the fenestrae-associated cytoskeleton ring, sieve plate associated cytoskeleton, fenestrae forming center, and defenestration-associated center [30, 32, 75–81]. Agents that disrupt actin such as cytochalasin D and latrunculin A increase the number of fenestrations, usually in the order of two-fold, associated with a marked reduction in sieve plates [13, 95, 104, 104, 135].

The key role of calcium in regulating fenestrations through effects on the cytoskeleton was reported by Gatmaitan et al [71]. Several agents were identified that reduced the diameter of fenestrations by about 20% in rat LSECs. All were associated with an increase of intracellular calcium by two-three folds. Agents that reduced fenestration diameter included serotonin, metoclopramide, propranolol, indomethacin and calcium ionophore while agents with no activity included verapamil, diltiazem, nifedipine, ketanserin, imipramine, mianserin, pertussis toxin and dexamethasone. Calcium channel blockers (diltiazem, verapamil and nifedipine) and the calcium chelator, EGTA reversed the effect of serotonin. In addition, the increase of calcium induced by serotonin was linked with phosphorylation of myosin light chain and reduced levels of cAMP [71]. In another study, serotonin reduced the diameter of fenestrations by 20% associated with an increased thickness of the fenestrae-associated actin ring by 6 nm, confirming the interaction between serotonin, actin and fenestrations [82]. More recently the selective serotonin receptor agonist, 22,5-dimethoxy-4-iodoamphetamine (DOI) has been shown to be a potent modulator of fenestrations through VEGF mediated mechanisms [18, 83].

In endothelial cells, VEGF activates cell division, angiogenesis and vascular permeability. VEGF increases intracellular calcium, phosphorylates myosin light chain and causes retraction of the cytoskeleton [84, 85]. VEGF generates fenestrations and caveolae in a number of different endothelial cells included tumor [86], renal [87] and adrenal [88] endothelial cells. In the liver, hepatocytes produce VEGF which acts of liver endothelial cells via the receptors: VEGFR-1 (Flt-1) and VEGFR-2 (KDR/Flk-1) of which VEGFR-2 is the most important [28, 89, 90]. VEGF is expressed more highly in the pericentral regions reflecting hypoxia which is the primary stimulus for VEGF production, whereas VEGFR-2 is found along the entire sinusoidal endothelium [91].

In isolated liver endothelial cells, VEGF increases porosity about twofold, mostly through its effects on the number of fenestrations [28, 92]. VEGF converts punctuate caveolin-1 staining to aggregates of staining, the majority of which are located at the periphery of the LSECs. VEGF did not change total caveolin-1 protein expression. Indeed caveolin-1 labeling might eventually appear reduced by the redistribution of caveolin-1 onto the markedly increased number of fenestrations [93].

Systemic VEGF exposure generated by VEGF-expressing CHO cells implanted into nude mice stimulated mitosis and proliferation of liver endothelial cells and led to increased complexity and branching of sinusoids [89]. Conversely, transgenic inhibition of VEGF receptors altered the hepatic endothelium of early postnatal mice, including loss of endothelial lining in many sinusoids [94] and was associated with defenestration and hyperlipidemia [95], this integral role of VEGF in fenestration development has also been confirmed in the kidney [96]. VEGF is considered to be the major cytokine involved in the regulation of fenestrations [87].

Members of the Rho-like GTPase family also regulate the actin cytoskeleton in endothelial cells and are critical for membrane fusion [97]. Inhibition of the Rho pathway by C3-transferase caused reduction of myosin light chain phosphorylation, loss and retraction of actin filaments and led to increased porosity and the formation of large gaps. Activating Rho with lysophosphatidic acid increased myosin light chain phosphorylation and actin filaments and led to defenestration [98].

Other factors that influence fenestrations, presumably via actions on actin are endothelin 1 and nitric oxide. Endothelin 1 increased intracellular calcium and decreased fenestration diameter whereas prostaglandin decreased intracellular calcium and increased fenestration diameter [99]. In another study endothelin 1 decreased diameter of fenestrations from 123 nm to 46 nm, an effect abolished by blockade of the ET_B_-R, and only partially abolished by ET_A_-R antagonism [100]. Antagonism of the ET_A_-R caused a marked increase in fenestration diameter associated with gap formation [101]. Nitric oxide is involved in the maintenance of fenestrations. Caveolin-1 and endothelial nitric oxide co-locate in the cell membranes lining fenestrations and caveolin-1 is attached to actin [102]. Activation of calmodulin by increased levels of intracellular calcium releases endothelial nitric oxide synthase from caveolin-1, thereby increasing production of nitric oxide [93]. Importantly, it has been shown that the effects of VEGF on the phenotype of LSECs require autocrine production of nitric oxide [69].

On the basis of some studies showing the expression of caveolin-1 in fenestrations and the similarity in their dimensions and appearances, it had been proposed that fenestrations are a form of caveolae and that caveolin-1 is a possible marker [45, 93]. While the role of caveolin 1 in the formation of diaphragmed fenestrations of the kidney glomerular endothelium had already been ruled out [37], we investigated its role in the non-diaphragmed fenestrations in the liver using caveolin-1 knockout mice and performed electron microscopic immunogold caveolin-1 staining in wild type mice. Fenestrations were normal in the knockout mice and caveolin-1 did not decorate fenestrations in wild type mice. Therefore undiaphragmed and diaphragmed fenestrations are unlikely to be caveolae and do not require caveolin-1 for their formation [103]. In addition, PV-1 had been identified as marker of fenestrations, but has recently been shown to be required for diaphragm formation on diaphragmed fenestrations, but is not present in the LSEC at all [104].

Fenestrations form as a result of fusion of opposing plasma membranes [79, 105–107] by a process of membrane or pore fusion [108]. In other cell types, this process generates membrane pores with diameters of 100-500 nm [109] and is highly sensitive to actin-modifying agents [97]. The actin cytoskeleton prevents the development of protein-free patches in membranes and subsequent contact of protein-free membranes domains that are required for membrane fusion to be initiated (‘actin barrier’). Actin is then involved in the subsequent development and stabilization of fusion pores [97, 108]. Thus there is a biphasic dose-dependent response to actin modifying agents with low doses stimulating fusion and high doses inhibiting fusion [97]. It has been proposed that actin depolymerization is initially required to allow membranes to dock, whereas the final membrane fusion process requires the re-establishment of an actin network [97]. The time for the opening of a fusion pore in other cells is less than 20 minutes [109] which provides some estimate of the rate of fenestration formation. In LSECs, actin reorganization might allow more cell membrane fusion to be initiated by removing the intervening actin barrier, leading to increased fenestrations. This is consistent with the observation that actin disruptors cause increased fenestrations and decreased sieve plates. On the other hand, a different level of actin reorganization will impede the subsequent completion of membrane fusion, causing defenestration. The effects may also be time-dependent and many studies showing increased fenestration were measured over longer periods than those showing reduced fenestrations. The role of various fusion proteins (eg SNAREs, Rabs, dynamin) [108, 110] and other pathways involved in the regulation of membrane fusion [97, 108] have not been investigated with respect to fenestration formation. However, a complete peristomal ring of sterols, considered to contribute to membrane fusion, has been detected lining the rim of fenestrations, but not around gaps [107]. This was one of the first indications that the cell membrane, its composition and the arrangement of these components may be key in fenestration formation and maintenance.

## The Sieve-Raft Hypothesis

The cell membrane and its distinct and specialized regions known as microdomains determine myriad biological processes including cell signalling, protein trafficking, cell viability, and cell movement. Recently we have shown that membrane microdomains, also known as lipid raft and non- raft regions, also regulate fenestrations [19]. This work has been described as a “*a major advance in our understanding”* of the mechanisms that regulate the formation of sieve plates and fenestrations” [111]. Lipid rafts are a distinct type of membrane microdomains that are enriched in sphingolipid, cholesterol and protein. They vary in size from 10-200 nm, and may aggregate to form micrometre-sized structures [112]. Sphingolipids and cholesterol engender membrane stability and provide a platform for many membrane proteins such as membrane receptors. Rafts are tethered to the actin cytoskeleton through protein complexes such an ezrin-radixin-moesin and stabilin which have a pivotal role in maintaining their structure and integrity [113, 114]. The size of individual membrane rafts, like that of fenestrations, is below the limits of resolution of light microscopy and their visualization with fluorescence microscopy has had limited success. While the presence and localisation of rafts has suffered from important controversies due to isolation methodologies and the inability to reliably visualize them, major technological advances in microscopy such as Structured Illuminated Microscopy (SIM), lipidomic and proteomic platforms such as mass spectrometry and development of model systems are leading advances in the field [112]. It is now widely accepted that assemblies of sphingolipids, cholesterol and proteins into raft platforms, or liquid- ordered phases of the membrane, and their corresponding liquid disordered phase non- raft neighbours and the patterns of phase segregation that occur, are vital for signalling, membrane vesiculation, trafficking and viral infection.

**Figure 2:**
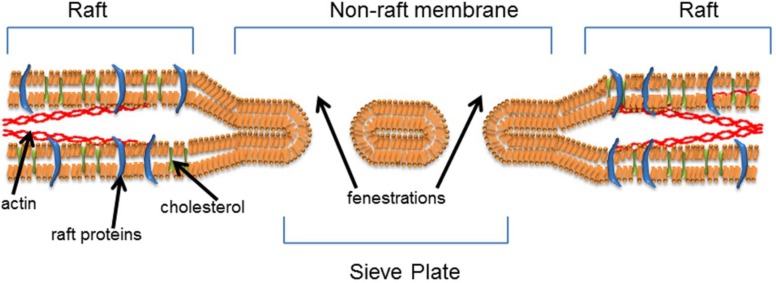
The Sieve-Raft hypothesis: the composition and arrangement of lipids in the cell membrane is paramount in determining fenestration formation and cell function. We propose that fenestrations form in non-raft microdomains of the lipid bilayer and that rafts and actin engender membrane stability, while limiting fenestration formation.

In 2010, in collaboration with our colleagues at UC Davis, we utilised SIM to resolve the topography of fenestrations and sieve plates and for the first time show a detailed three-dimensional map of their structure [32]. When we stained the plasma membrane of the LSEC, we noted discrete membrane structures that were intercalated between the sieve plates. On the basis of their size and appearance we postulated that these structures are membrane rafts and potentially involved in the regulation of sieve plates. In order to test this hypothesis, we then applied 3D-SIM, Total Internal Refractive Fluorescence Microscopy (TIRFM) and Scanning Electron Microscopy (SEM) techniques to isolated LSECs and visualized membrane rafts, fenestrations and actin under various conditions [19]. These studies indicated that there is a clear inverse distribution between fenestrations and membrane rafts and that fenestrations form in non- raft regions of LSECs once the membrane-stabilizing effects of actin cytoskeleton and membrane rafts are diminished (Figure 1). We termed this the ‘sieve-raft hypothesis’ (Figure 2).

The final step in the formation of fenestrations requires the juxtaposition of the apical and basolateral membranes in very thin areas of cell cytoplasm. This process of cell membrane bending and fusion requires ATP and large-scale deformations of the lipid bilayers [13]. Recently it has been shown that plasma membrane fusion can only occur when lipid rafts are depleted [113]. Further it has been shown that membrane fusion and pore formation is restricted by a dynamic resistance of the actin network in experimental membrane fusion models [74], suggesting that the formation of fenestrations requires retraction and or rearrangement of the normal sub-membrane actin cytoskeleton. We propose that the final process leading to the formation of fenestrations may be similar to the generation of membrane vesicles, which also requires disruption of the actin cytoskeleton and are associated with increased lipid-disordered, non- raft microdomains [115]. Vesiculation occurred spontaneously in membranes when line tension associated with rafts was reduced and the tethering by actin cytoskeleton released. This is consistent with our observation that small pores are seen adjacent to fenestrations in the non- raft microdomains of the LSEC. Very recently, the splitting apart (fission) of membranes, an essential step prior to apical and basolateral membrane fusion, has been shown to be dependent on dynamin, GTP release and Phosphatidylinositol 4,5-bisphosphate (PIP2) localisation. PIP2 is a phospholipid most enriched in non-raft microdomains of the cell membrane [110].

The raft-sieve hypothesis is the synthesis of our recent findings and current knowledge on membrane biology and outlines that transcellular fenestrations form in phase segregated, non- raft domains of the plasma membrane (Figure 2). These domains can be uniquely identified by their lipid and protein species which impart unique biophysical properties to the membrane, providing the microenvironment in which fenestrations can form. Cell plasma membrane curvature, deformation, vesiculation and elasticity are core fields for cell membrane research. Regulation of these properties are essential steps in many fundamental cell processes such as endocytosis [116], intercellular nanotube formation [117], red blood cell deformation for blood flow through capillaries [118], ovum fertilisation by sperm in meiotic reproduction [119] as well as the focus of our own research on fenestrations in the LSEC. Underlying these processes is the structural contribution of the lipid and protein content on the plasma membrane.

High cholesterol and sphingolipid components have been shown to engender membrane stability and reduced elasticity, and reduction in the concentration of these molecules leads to an increased capacity for membrane curvature [118]. The lipid content of bacterial membranes has been specifically shown to alter in order to induce membrane curvature in helices formation [120]. Definition of these fundamental biological interactions in other cells has led to powerful insight into viral and bacterial infection of cells and reproduction, yet the lipid and protein properties of the biologically essential fenestrated cell membrane are unknown.

We believe that the Sieve-Raft hypothesis may underlie fenestration formation in all fenestrated cells and suggest that while more experimental data is needed, the next step forward in greater understanding of the structural biology of fenestrations is probing cell membranes. Identification and localisation of the component lipids and proteins, and interrogation of the interactions of these constituents and how they form fenestrations requires technologies and tools not previously applied to study fenestrations. An innovative correlative approach using cutting edge lipidomics, proteomics and visualisation will provide detailed information regarding cell membrane biology and how specific patterns of interaction between lipids and proteins can result in the unique biological phenomena that is cellular fenestrations.
